# Sulforaphane Potentiates RNA Damage Induced by Different Xenobiotics

**DOI:** 10.1371/journal.pone.0035267

**Published:** 2012-04-23

**Authors:** Carmela Fimognari, Monia Lenzi, Piero Sestili, Eleonora Turrini, Lorenzo Ferruzzi, Patrizia Hrelia, Giorgio Cantelli-Forti

**Affiliations:** 1 Department of Pharmacology, Alma Mater Studiorum – University of Bologna, Bologna, Italy; 2 Dipartimento di Scienze Biomolecolari - Università degli Studi di Urbino “Carlo Bo”, Urbino, Italy; Wayne State University School of Medicine, United States of America

## Abstract

**Background:**

The isothiocyanate sulforaphane (SFN) possesses interesting anticancer activities. However, recent studies reported that SFN promotes the formation of reactive oxygen species (ROS) as well as DNA breakage.

**Methodology/Principal Findings:**

We investigated whether SFN is able to damage RNA, whose loss of integrity was demonstrated in different chronic diseases. Considering the ability of SFN to protect from genotoxicity, we also examined whether SFN is able to protect from RNA damage induced by different chemicals (doxorubicin, spermine, S-nitroso-N-acetylpenicillamine, H_2_O_2_). We observed that SFN was devoid of either RNA damaging and RNA protective activity in human leukemic cells. It was able to potentiate the RNA damage by doxorubicin and spermine. In the first case, the effect was attributable to its ability of modulating the bioreductive activation of doxorubicin. For spermine, the effects were mainly due to its modulation of ROS levels produced by spermine metabolism. As to the cytotoxic relevance of the RNA damage, we found that the treatment of cells with a mixture of spermine or doxorubicin plus SFN increased their proapoptotic potential. Thus it is conceivable that the presence of RNA damage might concur to the overall toxic response induced by a chemical agent in targeted cells.

**Conclusions/Significance:**

Since RNA is emerging as a potential target for anticancer drugs, its ability to enhance spermine- and doxorubicin-induced RNA damage and cytotoxicity could represent an additional mechanism for the potentiating effects of SFN associated with anticancer drugs.

## Introduction

Broccoli and broccoli sprouts contain wide amounts of glucosinolates [1]. Numerous studies have demonstrated the chemopreventive effect of increasing cruciferous vegetable intake against cancer, which is mainly imputable to the activity of various isothiocyanates, highly biologically active compounds formed upon enzymatic hydrolysis of glucosinolates [2]. Sulforaphane [SFN, 1-isothiocyanato-4-(methyl-sulfinyl)-butane; CH3-SO-(CH2)4-N C S], a well characterized isothiocyanate compound, was found to be obtained from glucoraphanin, a major glucosinolate in broccoli/broccoli sprouts [3]. The chemopreventive properties of SFN against cancer are through both “blocking” and “suppressing” effects [2]. The blocking function of SFN is achieved through inducing phase 2 detoxification enzymes that promote excretion of carcinogens [2]. Subsequent studies revealed the suppressing effects of SFN mediated by its pleiotropic capacity to simultaneously modulate multiple cellular targets involved in cell proliferation and apoptosis [4]. The ability of SFN to induce apoptosis and cell-cycle arrest is associated with regulation of many molecules including Bcl-2 family proteins, p53, caspases, p21, cyclins, and cyclin-dependent kinases [4]. SFN was also shown to suppress angiogenesis and metastasis by the downregulation of vascular endothelial growth factor, HIF-1α, matrix metalloproteinase-2 and matrix metalloproteinase-9 [4].

Genomic DNA breaks represent an important trigger of apoptosis [5]. Accumulating evidence has shown that SFN increases intracellular reactive oxygen species (ROS) levels and induces apoptosis in various cancer cell lines [6]–[8]. Although the antitumorigenic effect of SFN is well established, a recent study demonstrated that SFN promoted intracellular ROS formation as well as DNA breakage in two different cell types [9]. The formation of DNA single strand breaks was clearly demonstrated in cells exposed to supranutritional concentrations of SFN. On the contrary, no sign of DNA lesions or micronuclei induction could be observed at the nutritionally attainable concentrations of SFR (≤10 µM) [9], [10].

In the present study, we performed our investigation to see whether SFN is able to target and damage RNA. We used nutritional and supranutritional concentrations of SFN.

RNA may be more susceptible to damaging agents than DNA for different reasons. RNA is indeed mostly single-stranded and its bases are neither protected by hydrogen bonding nor located inside the double helix [11]. Almost all of the cellular RNA has functional capacity for protein synthesis, whereas only 5% of the transcribed sequences of genomic DNA encode proteins [12]. Finally, RNA is more abundant than DNA. In this view, it is highly probable that significant damage to RNA occurs when cells are exposed to nucleic acids damaging agents.

Despite its potential to affect cell physiology, potential triggers of RNA damage as well as its pathophysiological implications remain largely unknown. A significant loss of RNA integrity has been demonstrated in advanced human atherosclerotic plaques [13], [14]. Oxidative RNA damage has been described in several neurodegenerative diseases including Alzheimer disease, Parkinson disease, dementia with Lewy bodies, and prion diseases [15]–[17]. Thus, further studies on RNA damage and its surveillance may have a significant impact on the understanding of the pathophysiology of currently unresolved complex diseases.

Taking into account the demonstrated ability of SFN to protect cells from genotoxic insult [18], [19], we also investigated whether SFN is able to protect cells from RNA damage induced by different chemicals and its mechanism of action.

## Methods

### Ethics Statement

Blood donors provided written, informed consent for the study use of the samples at the time of donation. The described study was approved by the Comitato Etico dell'Azienda Unità Sanitaria Locale di Bologna.

### Chemicals

Reagent grade chemicals were purchased from Sigma (St. Louis, MO, USA). SFN (Sigma) was dissolved in DMSO. The stock solution (50 mM) of S-nitroso-N-acetylpenicillamine (SNAP) was prepared by combining equal volumes of N-acetyl-D-penicillamine (19 mg/mL in 100% ethanol) and NaNO_2_ (7 mg/mL in RNase free water). The mixture was acidified with 50 µL of hydrochloric acid (19% v/v) *per* 1 mL of SNAP solution and incubated for at least 30 min at 4°C before use. The stock solution was prepared immediately before administration.

### Lymphocyte isolation

Human peripheral blood (60 mL) was obtained from normal healthy volunteers of AVIS (Italian Association of Voluntary Blood Donors). Human mononuclear cells were isolated by density gradient centrifugation using Histopaque-1077 (Sigma). Lymphocytes at a concentration of 4×10^5^ cells/mL were added with 10 µg/mL of phytohemagglutinin (Sigma).

### Cell culture

Human leukemia Jurkat (acute T lymphoblastic leukemia) and HL-60 (acute promyelocytic leukemia) cell lines were purchased from Istituto Zooprofilattico Sperimentale Lombardia ed Emilia Romagna, Brescia, Italy). KU812F (chronic myeloblastic leukemia) cell line was purchased from LGC Standards (Sesto S. Giovanni, Italy). Jurkat, HL-60, KU812F cell lines and non-transformed human T lymphocytes were grown in suspension and propagated in RPMI 1640 supplemented with 10% (Jurkat, KU812F, T lymphocytes) or 20% (HL-60) heat-inactivated bovine serum, 1% antibiotics (all obtained from Sigma). To maintain exponential growth, the cultures were divided every third day by dilution to a concentration of 1×10^5^ cells/mL.

### Cell treatments

Cells were treated with different concentrations of SFN (0–30 µM) for 6 or 24 h at 37°C. The RNA-damaging compounds used in this study were SNAP, spermine NONOate, doxorubicin, and H_2_O_2_ (all obtained from Sigma). Cells were treated with different concentrations (0.0–0.5 mM) of SNAP, spermine NONOate, or doxorubicin for 24 h at 37°C. An additional time point (6 h) was included for spermine. For H_2_O_2_, the protocol was slightly modified. The cultures were treated with different concentrations of H_2_O_2_ (0.0–0.5 mM) in PBS for 6 h at 37°C. The range of concentrations of the potential RNA-damaging agents were selected considering the quantity of total RNA extracted *per* cell, as recently suggested [14], [20], [21].

For assessing the potential protective activity of SFN, two different treatment protocols were used:

Pre-treatment protocol: the cells were incubated for 24 h with SFN, then were washed and treated with the RNA toxic compounds for 24 h (6 h for H_2_O_2_).
Co-treatment protocol: the cultures were incubated for 24 h with SFN and the RNA toxic compounds (6 h for H_2_O_2_).

The same experimental protocols were used in cell-free systems, where RNA was extracted and then treated with the different compounds in RNase-free water, and in HL-60, KU812F and normal human T cells.

### Cell viability

Viability was determined immediately after treatments by using an EasyCyte 5HT flow cytometer (Millipore, Guava Technologies, Hayward, CA, USA), according to the manufacturer's recommendations. Briefly, cells were mixed with an adequate volume of Guava ViaCount Reagent (Millipore) and allowed to stain for at least 5 min at room temperature. The Guava ViaCount Reagent provides absolute cell count and viability data based on the differential permeability of DNA-binding dyes and the analysis of forward scatter. The fluorescence of each dye is resolved operationally to allow the quantitative assessment of both viable and non-viable cells present in a suspension.

### Extraction of RNA

After cell treatment, RNA was isolated with an Agilent Total RNA isolation Mini Kit (Agilent Technologies, Palo Alto, CA, USA), according to the manufacturer's recommendations. Considering the high susceptibility of RNA to RNase, all the steps were performed by using nuclease-free water, RNase-free final collection tubes and RNase inhibitors. Briefly, 350–400 µL of lysis solution were added to cell pellet and the cell homogenate was centrifuged through a mini-prefiltration column. The flow-through was mixed with an equal volume of 70% ethanol, incubated for 5 min at room temperature and centrifuged through a mini-isolation column. The flow-through was discarded and the RNA-loaded column was transferred into an RNase-free final collection tube. Then, the purified RNA was eluted by addition of 10 µL of nuclease-free water.

### Analysis of RNA damage

RNA analysis was performed by microfluidic capillary electrophoresis with the Agilent 2100 bioanalyzer. Tiny amounts of RNA samples are separated in the channels of the microfabricated chips according to their molecular weight and subsequently detected via laser-induced fluorescence detection. The result is visualized as an electropherogram where the amount of measured fluorescence correlates with the amount of RNA of a given size. A software algorithm then allows the calculation of an RNA Integrity Number (RIN). The RIN algorithm is based on a selection of informative features from the electropherograms. For this purpose, each electropherogram is divided into the following nine adjacent segments covering the entire electropherogram: a pre-region, a marker-region, a 5S-region, a fast-region, an 18S-region, an inter-region, a 28S-region, a precursor-region, and a post-region. In addition, several global features are extracted, i.e. features that span several segments. Among these, the average and maximum height, areas and their ratios, total RNA ratio and the 28S area ratio are the most important. The gradual degradation of RNA is reflected by a continuous shift towards shorter fragment sizes. For classification of RNA integrity, ten categories are defined from 0 (totally degraded RNA) to 10 (fully intact RNA) [22].

### ROS detection

2′,7′-dichlorofluorescein-diacetate (DCFH-DA) was used for ROS detection. DCFH-DA is cleaved intracellularly by nonspecific esterases to form 2′,7′-dichlorodihydrofluorescein (DCFH), which is further oxidized by ROS to form the highly fluorescent compound 2′,7′-dichlorodihydrofluorescein (DCF) [23]. Briefly, 1.5×10^6^ cells were pre-treated with SFN 10 µM for 3 or 24 h. Then, samples were washed, treated with spermine 0.5 mM and additionally incubated for 3 or 24 h. At the end of the treatment, the samples were washed and 0.5×10^6^ cells were stained with DCHF-DA 5 µM. After 20 min of incubation at 37°C, fluorescence intensity was monitored at 510 nm using VICTOR 3 V Multilabel Counter (PerkinElmer, Waltham, MA, USA). Data were expressed as percentage of the control (untreated cells).

### Measurement of thioredoxin reductase activity

The thioredoxin reductase activity was determined by the colorimetric assay based on the reduction of 5,5′-dithiobis(2-nitrobenzoic) acid (DTNB) with NADPH to 5-thio-2-nitrobenzoic acid (TNB), which produces a strong yellow color that is measured at 412 nm by VICTOR 3 V Multilabel Counter (PerkinElmer). Briefly, 1.5×10^6^ cells were treated with SFN 10 µM for 3 h or 24 h. In order to determine the DTNB reduction due only to the thioredoxin reductase activity present in the samples, two assays were performed. The first assay measured the total DTNB reduction by the sample and the second one measured the DTNB reduction by the sample in the presence of the thioredoxin reductase inhibitor solution (Sigma). The difference between the two analyses represents the DTNB reduction due to thioredoxin reductase activity alone. The results were expressed in U/mL and normalized to protein concentration determined by Bradford reagent (Sigma). Thioredoxin reductase (Sigma) was used as positive control.

### Analysis of GSH levels

The level of total glutathione [*i.e.* glutathione (GSH) and glutathione disulfide (GSSG)] was measured by the Gluthatione Assay Kit (Sigma), in which catalytic amounts (nmoles) of GSH caused a continuous reduction of DTNB to TNB, spectrophotometrically measured at 412 nm by VICTOR 3 V Multilabel Counter (PerkinElmer). The GSSG formed is recycled by glutathione reductase and NADPH. The rate of TNB production is proportional to the concentration of glutathione within the sample. Briefly, 1.5×10^6^ cells were pre-treated with SFN 10 µM for 3 h or 24 h. Then, samples were washed and treated with spermine 0.5 mM for additionally 3 h. At the end of the treatment, the samples were first deproteinized with a 5% 5-sulfosalicylic acid solution (Sigma), centrifuged to remove the precipitated protein and then assayed for total glutathione. The results were expressed as glutathione concentration (µM).

### Detection of apoptosis by flow cytometry

Flow cytometric procedures were performed with an EasyCyte 5HT flow cytometer. Approximately 5000 events (cells) were evaluated for each sample. In all cytofluorimetric determinations, cell debris and clumps were excluded from the analysis by gating. Experiments were conducted by using duplicate samples for each treatment, and each experiment was repeated at least three times. Cells were treated with 1) doxorubicin or SFN 10 µM plus doxorubicin for 24 h, or 2) SFN 10 µM for 24 h, washed and treated with spermine for 24 h. After 24 h, cells were washed and resuspended in drug-free culture medium for further 24 h. Aliquots of 2.0×10^4^ cells were stained with 100 µL of Guava Nexin Reagent (Millipore, containing ANNEXIN-V-phycoerythrin and 7-amino-actinomycin D) and incubated for 20 min at room temperature in the dark. Samples were then analyzed by flow cytometry.

### Statistical analysis

All results are expressed as the mean ± S.E. of at least three independent experiments. Differences among treatments were evaluated by ANOVA, followed by Bonferroni t-test, using GraphPad InStat version 3.00 for Windows 95 (GraphPad Software, San Diego, CA, USA). P<0.05 was considered significant.

## Results

**Figure 1:**
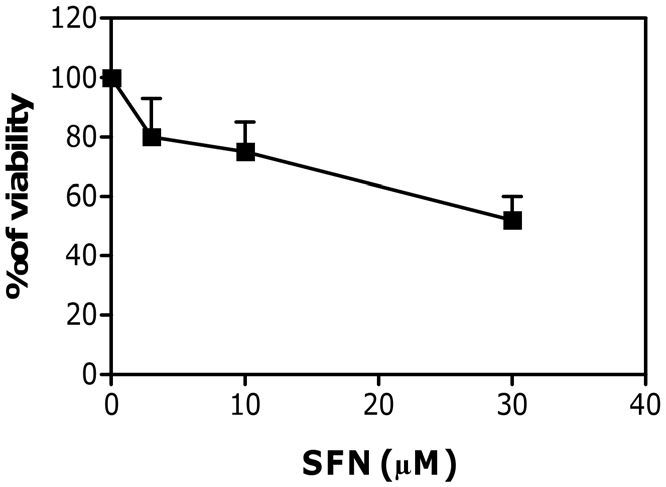
Effect of SFN on viability of Jurkat cells. The viability was determined immediately after treatments, as detailed in Section 2. Data are means ± SEM of three independent experiments.

To exclude that RNA fragmentation was an artifact associated with cell death, we first analyzed the effect of the treatment protocols on the cell viability. A general approach in performing genotoxicity test is to avoid the testing of doses that decrease viability, compared to the concurrent control cultures, by more than 60% [24]. Treatment of Jurkat cells with different concentrations of SFN slightly modified cell viability (Figure 1). At the highest dose tested (30 µM), SFN decreased cell viability by 48%. Similar results were recorded for the other cell lines (data not shown). The analysis of RNA-damaging activity was therefore performed until the concentration of 30 µM.

**Figure 2:**
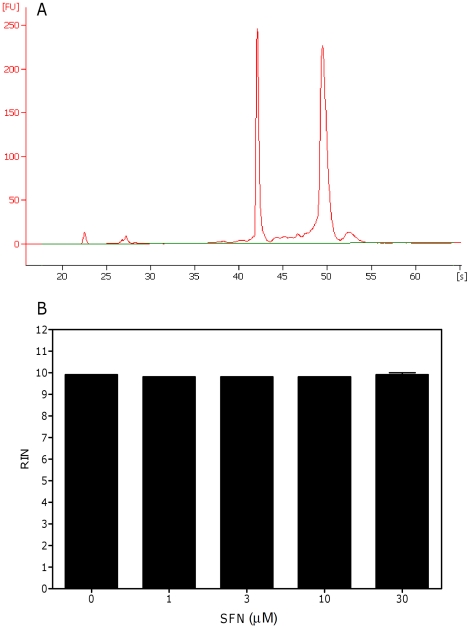
Effect of SFN on RNA integrity on Jurkat cell system. A representative electropherogram of RNA size distribution (A) and RIN values (B) calculated after cell treatment with SFN for 24 h. Data are means ± SEM of three independent experiments.

We then analyzed the ability of SFN to damage RNA. The RNA-damaging effect of SFN was assessed by RIN measurement both in cell systems and in cell-free systems. As shown in Figure 2A, SFN did not alter RNA integrity in cell systems. The electropherogram indeed contains the marker peak at about 24S as well as 3 prominent peaks corresponding to small RNAs, 18S, and 28S rRNA. The values of RIN reported at all the concentrations tested were similar to that observed in untreated cultures (9.8 *vs* 9.9, respectively) (Figure 2B). Similar values were observed in cultures treated with SFN for a shorter time (6 h) (data not shown).

**Figure 3:**
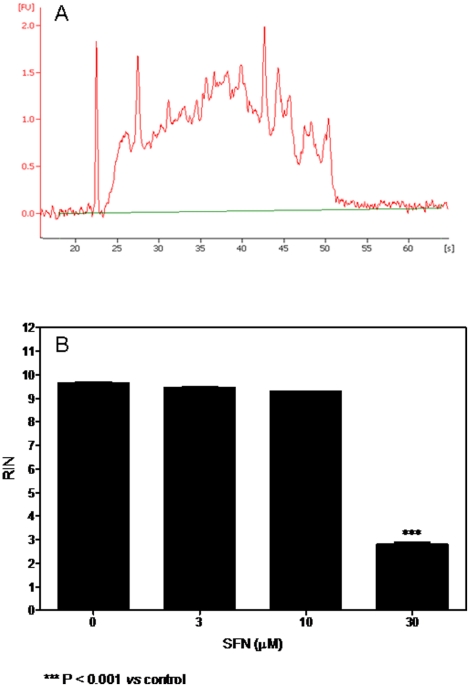
Effect of SFN on RNA integrity on Jurkat cell-free system. A representative electropherogram of RNA size distribution (A) and RIN values (B) after 3 h of treatment with SFN of a cell-free system. Data are means ± SEM of three independent experiments.

Interestingly, when SFN was tested in a cell-free system, it induced a significant RNA damage (Figures 3A and B). The values of RIN were 2.8 at 3 h and 6 h and 2.4 at 24 h. Lower concentrations of SFN did not induce RNA damage (Figure 3B).

In the second part of the study, we analyzed the ability of SFN to protect cells against the RNA-damaging activity of spermine, SNAP, doxorubicin, and H_2_O_2_.

The previously performed analysis of the cytotoxic potential of spermine, SNAP, doxorubicin, and H_2_O_2_ in Jurkat cells demonstrated that they did not induce a decrease in cell viability by more than 60% compared to the untreated cultures up to the highest concentration tested (0.5 mM) (data not shown). The viability of doxorubicin-treated cells was significantly decreased. The only concentration exhibiting a toxicity lower than 60% was 0.01 mM (data not shown). Concentrations of 0.5 mM for spermine, SNAP and H_2_O_2_, and 0.01 mM for doxorubicin were then used in the experiments aimed at evaluating the protective ability of SFN against RNA-damaging activity of the above reported compounds.

**Figure 4:**
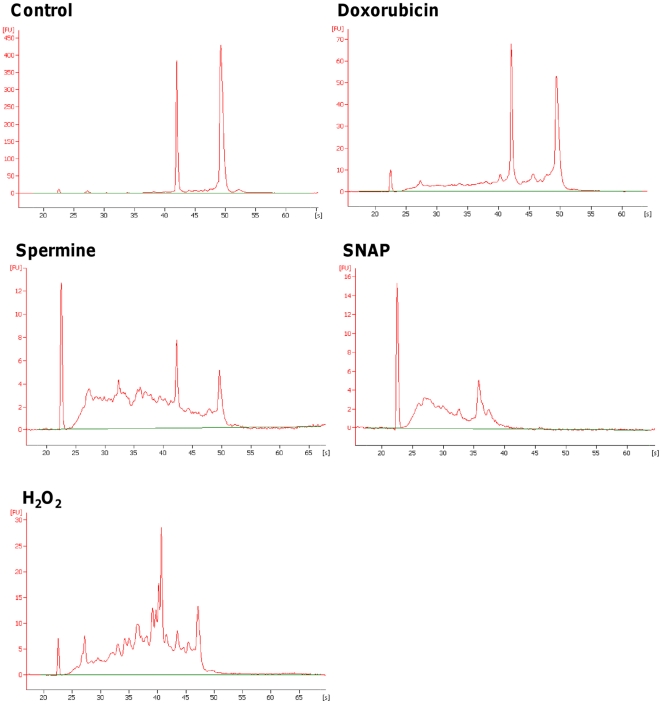
Effect of different xenobiotics on RNA integrity in Jurkat cells. Electropherograms of RNA size distribution recorded in untreated cultures and after 24 h of treatment with doxorubicin, spermine, SNAP or H_2_O_2_. The data are representative of three different experiments with similar results.

Spermine, SNAP, H_2_O_2_, and doxorubicin induced a pronounced RNA damage in Jurkat cells (Figure 4). The RIN values were 6.3±0.2 for doxorubicin, 5.5±0.3 for spermine, 2.1±0.2 for SNAP and 4.7±0.3 for H_2_O_2_ *vs* 9.6±0.3 for untreated cultures. Similar values were recorded in the other cell lines and in human lymphocytes. As an example, the RIN values were 7.0 and 5.1 for doxorubicin, 5.2 and 5.0 for spermine, 3.6 and 4.1 for SNAP and 6.0 and 5.0 for H_2_O_2_ in HL-60 cells and T lymphocytes, respectively.

**Figure 5:**
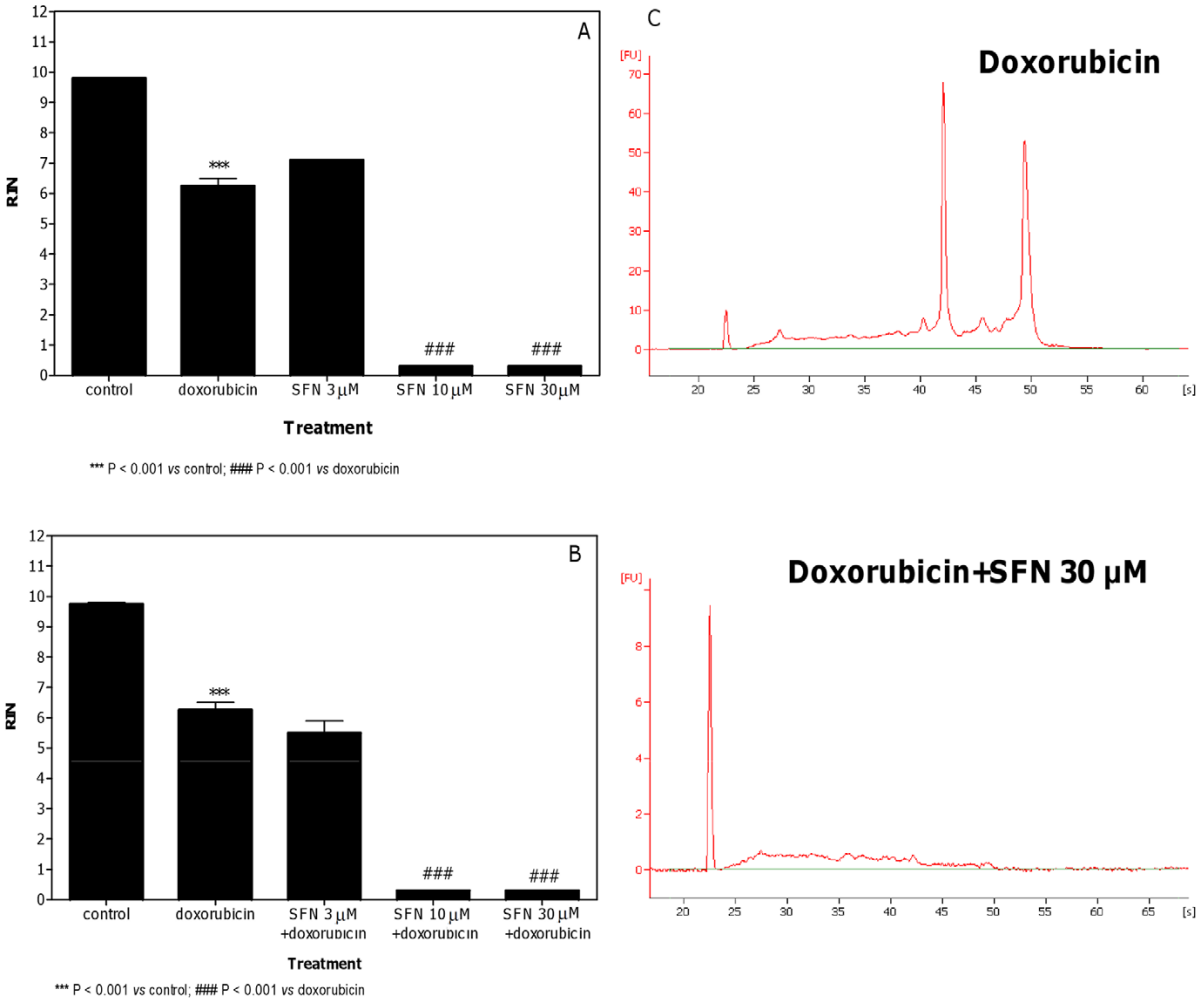
Effect of doxorubicin plus SFN on RNA damage in Jurkat cells. RIN values calculated after pre-treatment (A) or co-treatment (B) of cells with SFN plus doxorubicin and representative electropherograms of RNA size distribution after treatment with doxorubicin plus SFN (C). Data are means ± SEM of three independent experiments.

SFN produced a significant dose-related increase in the RNA damage induced by doxorubicin using both treatment protocols (Figure 5). In particular, SFN reduced the RIN recorded in the cultures treated with doxorubicin from 6.3 to values lower than 1. For both the pre-treatment and co-treatment protocols, the maximum reduction was obtained with SFN 30 µM, where a decrease in the heights of 18S and 28S peaks was well evident (Figure 5C).

**Figure 6:**
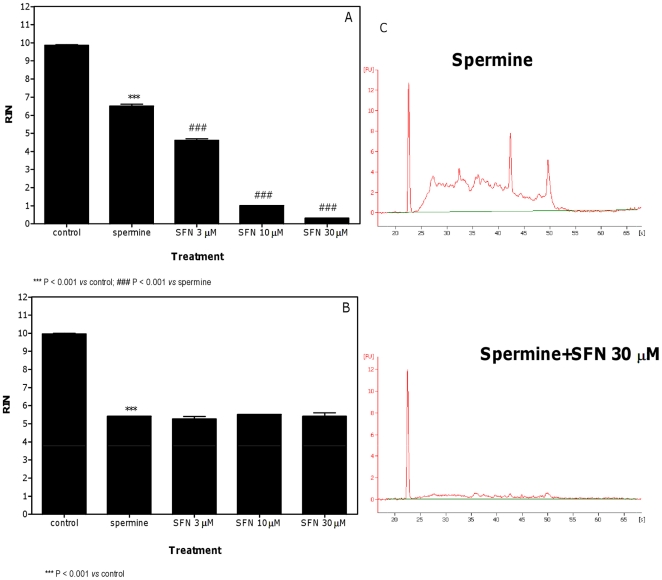
Effect of spermine plus SFN on RNA damage in Jurkat cells. RIN values calculated after pre-treatment (A) or co-treatment (B) of cells with SFN plus spermine and representative electropherograms of RNA size distribution after treatment with spermine plus SFN (C). Data are means ± SEM of three independent experiments.

Pre-treatment with all three SFN concentrations also reduced the RIN value induced by spermine (Figure 6A). The effect was clearly dose-dependent with the maximum effect at the concentration of SFN 30 µM, where an increase in the faint signals from cellular RNAs with a broad range of molecular weights was observed (Figure 6C). In the co-treatment protocol, SFN did not affect the RNA damage induced by spermine (Figure 6B).

**Figure 7:**
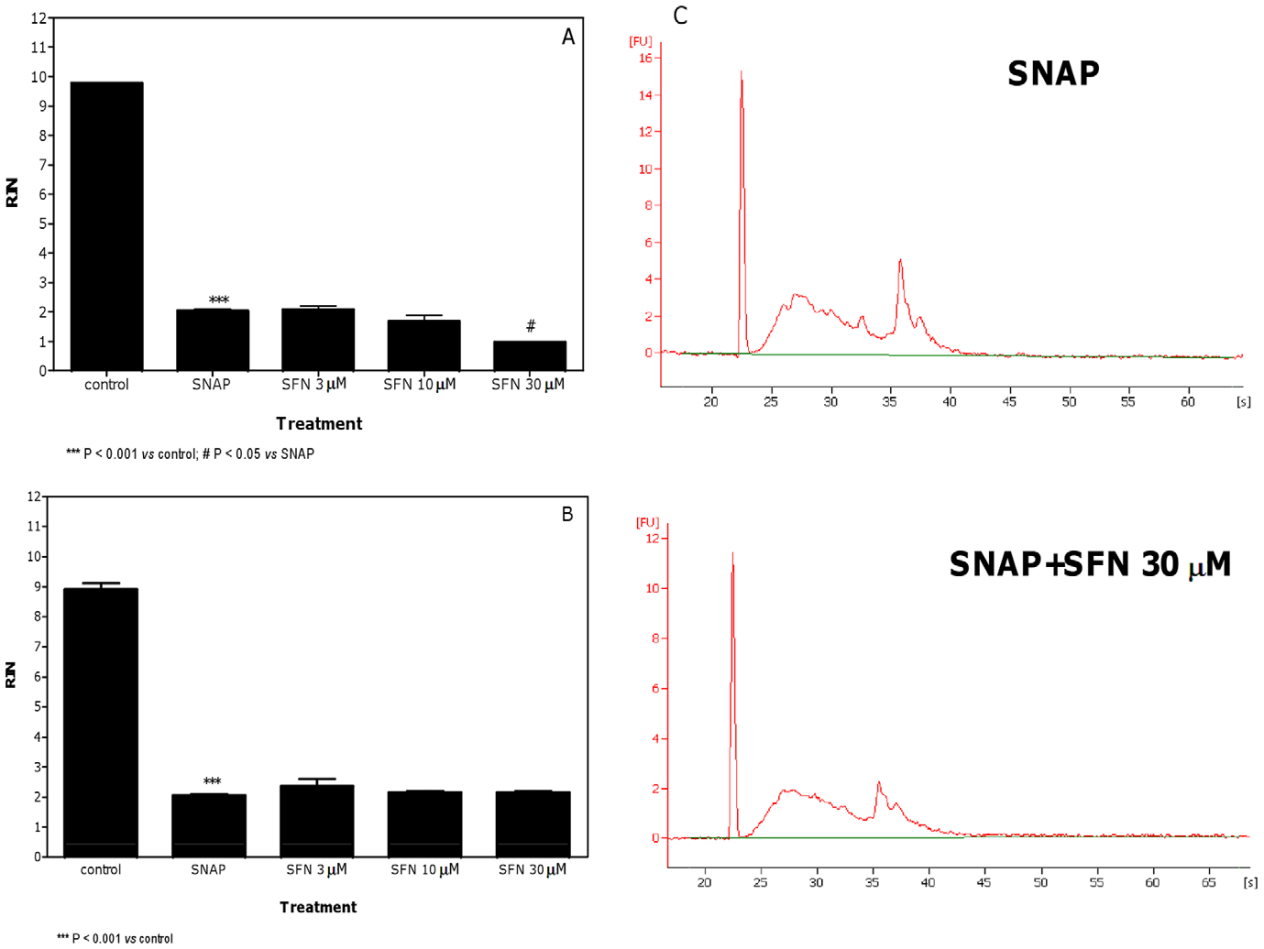
Effect of SNAP plus SFN on RNA damage in Jurkat cells. RIN values calculated after pre-treatment (A) or co-treatment (B) of cells with SFN plus SNAP and representative electropherograms of RNA size distribution after treatment with SNAP plus SFN (C). Data are means ± SEM of three independent experiments.

**Figure 8:**
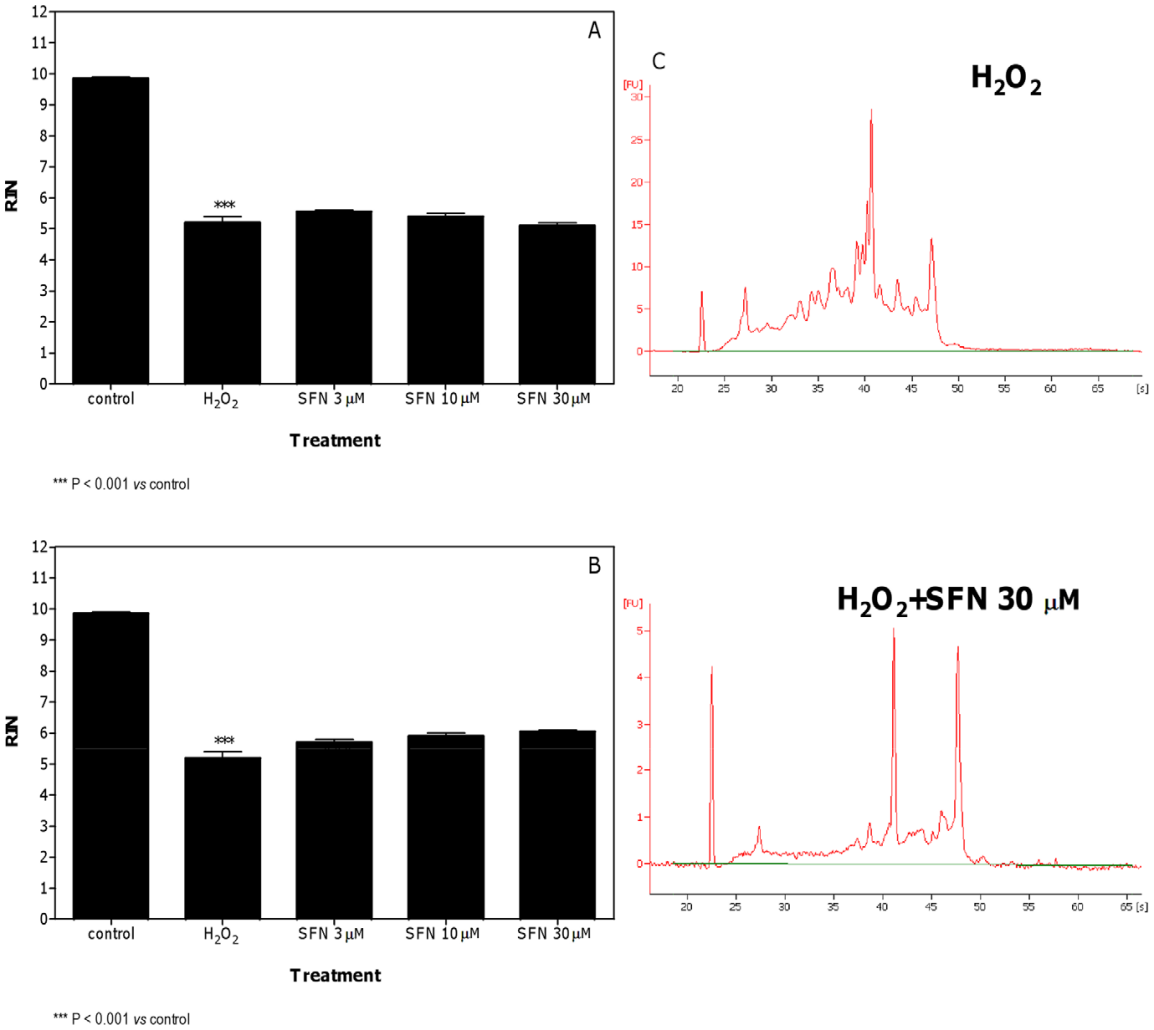
Effect of H_2_O_2_ plus SFN on RNA damage in Jurkat cells. RIN values calculated after pre-treatment (A) or co-treatment (B) of cells with SFN plus H_2_O_2_ and representative electropherograms of RNA size distribution after treatment with H_2_O_2_ plus SFN (C). Data are means ± SEM of three independent experiments.

The effect of SFN was negligible or null against the RNA damaging properties of SNAP and H_2_O_2_. For SNAP, only a slight decrease of RIN was observed in the pre-treatment protocol and at the highest concentration of SFN (Figures 7A and C). The values of RIN recorded in cells co-treated with SNAP plus SFN and in both treatment protocols for H_2_O_2_ were similar to those recorded in untreated cells (Figures 7B and 8).

**Figure 9:**
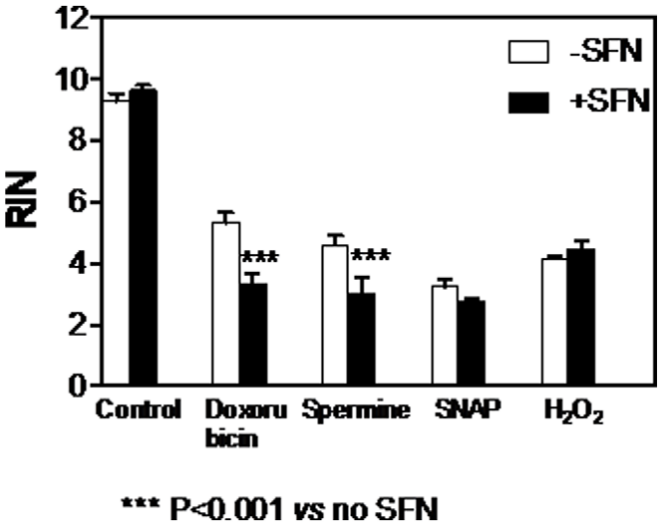
Effect of SFN 30 µM on RNA damage induced by doxorubicin, spermine, SNAP and H_2_O_2_ in HL-60 cells. RIN values were calculated after pre-treatment of cells with SFN for 24 h (6 h for H_2_O_2_). Data are means ± SEM of three independent experiments.

To study and compare the cell-line specificity of the effects of SFN on doxorubicin- and spermine-induced RNA damage, the same experiments were performed using different cell lines, namely HL-60 and KU812F. Results shown in Figure 9 indicate that SFN 30 µM was capable of potentiating the RNA damage induced by doxorubicin and spermine in HL-60 cells. No effect of SFN on the RNA damage induced by SNAP and H_2_O_2_ was recorded in the same cell line. Similar results were observed in KU812F cells and in normal T lymphocytes (data not shown), thus demonstrating a lack of cell-type specificity for these effects.

To test the possibility that the ability of SFN to enhance the RNA-damaging properties of doxorubicin and spermine was an artifact associated with a cytotoxic effect, we immediately analyzed the effect of the treatment protocols on Jurkat cell viability. No reduction of cell viability was observed after treatment with SFN plus doxorubicin or SFN plus spermine (data not shown).

On the whole, our results indicated a general inability of SFN to protect RNA from the insult caused by the four chemicals. Because of SFN's capacity to potentiate the RNA-damaging properties of doxorubicin and spermine, we performed a series of experiments in order to elucidate its mechanism of action in Jurkat cells.

**Figure 10:**
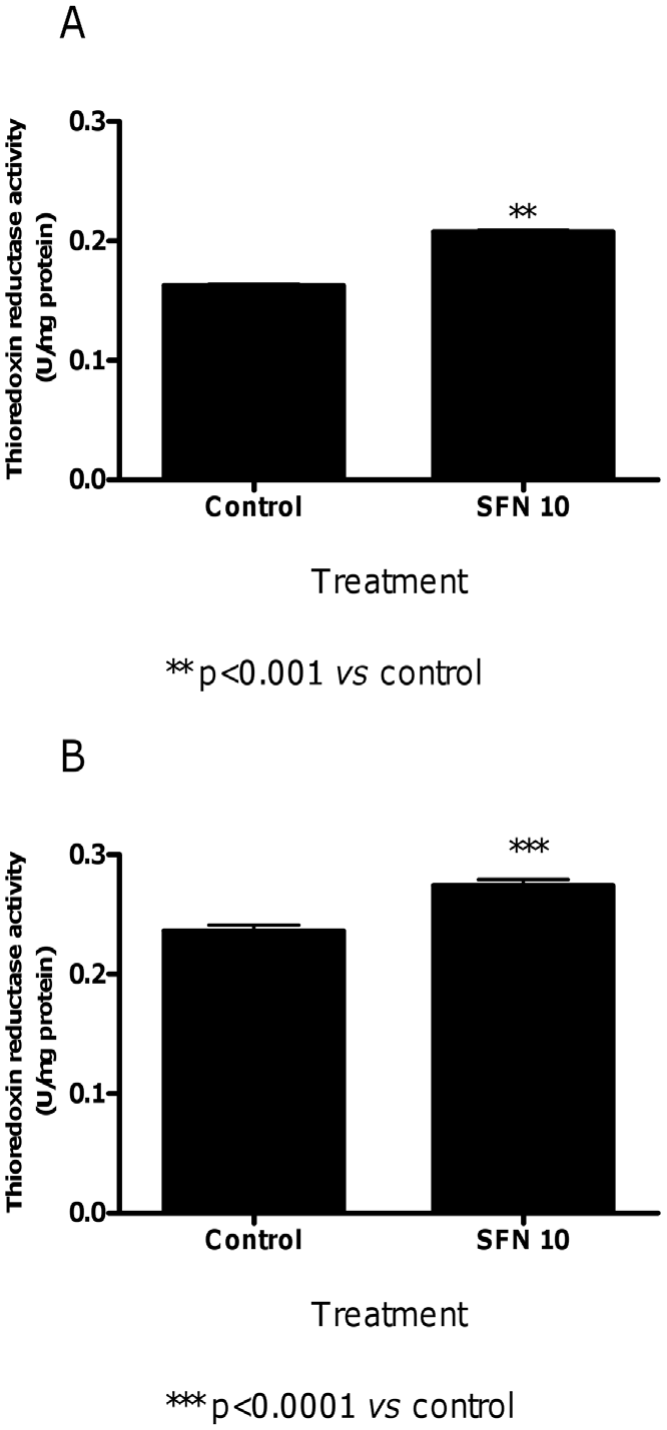
Effect of SFN on thioredoxin reductase activity in Jurkat cells. Effects of SFN (10 µM) on thioredoxin reductase activity after 3 h (A) or 24 h (B) of treatment. Data are means ± SEM of three independent experiments with triplicate dishes.

We first analyzed the ability of SFN to induce thioredoxin reductase activity. Treatment of cells with SFN 10 µM for 24 h significantly up-regulated thioredoxin reductase specific activity (Figure 10).

**Figure 11:**
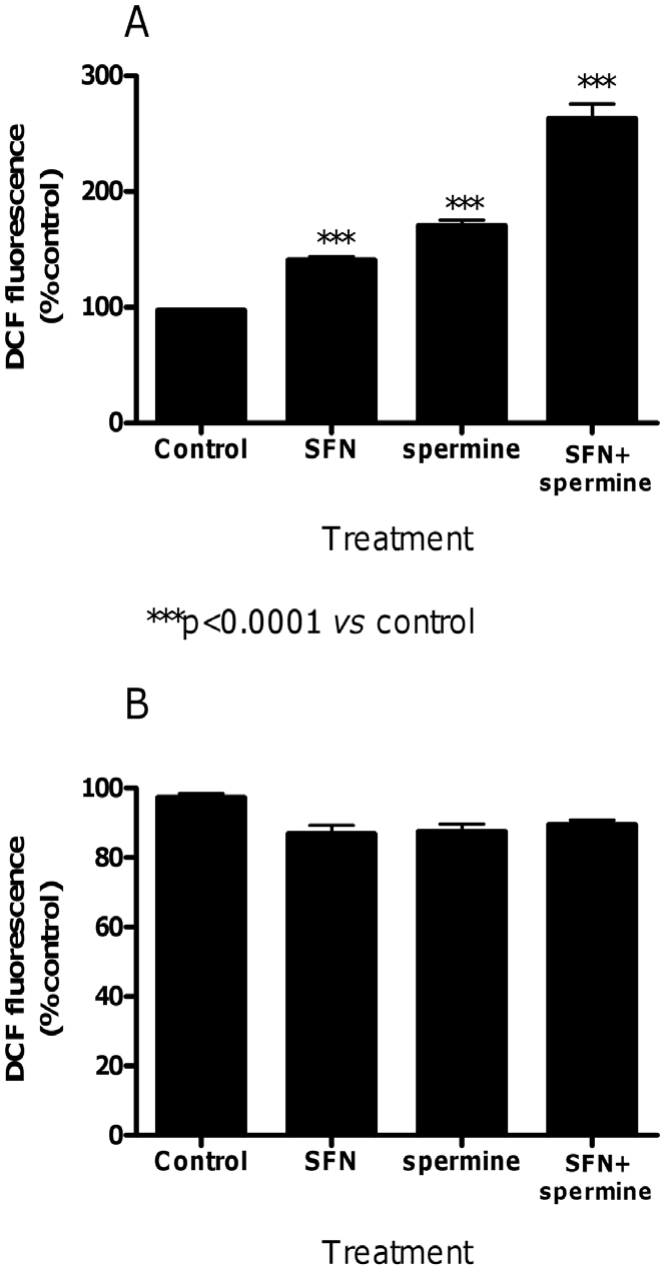
Effect of SFN and spermine on ROS levels in Jurkat cells. Effect of SFN, spermine and SFN plus spermine on ROS levels determined by microplate fluorescence reader. Cells were treated for 3 h (A) or 24 h (B) with SFN (10 µM), spermine (0.5 mM) or SFN (10 µM)+spermine (0.5 mM) to analyze the oxidation state of the cell by using DCFH-DA as fluorogenic probe. Results are expressed as percentages of control (untreated cells) and are means ± SEM of four independent experiments with triplicate dishes.

To know whether the effect of SFN on increasing the spermine-induced RNA damage was dependent on the increased formation of ROS, we determined the levels of ROS in cultures treated with SFN, spermine or SFN plus spermine (pre-treatment protocol). The formation of DCF was determined and quantified by means of a multilabel microplate reader. Results depicted in Figure 10A indicate that SFN (10 µM for 3 h) and spermine (0.5 mM for 3 h) caused the conversion of DCFH into its fluorescent by-product, a process which reflects the formation of ROS. Treatment of cells with SFN (10 µM for 3 h) plus spermine (0.5 mM for 3 h) greatly increased the formation of ROS. ROS formation was also investigated in cells treated with the above reported compounds for a longer exposure time (24 h). In this case, the levels of ROS were similar to those detected in untreated cells (Figure 11B).

**Figure 12:**
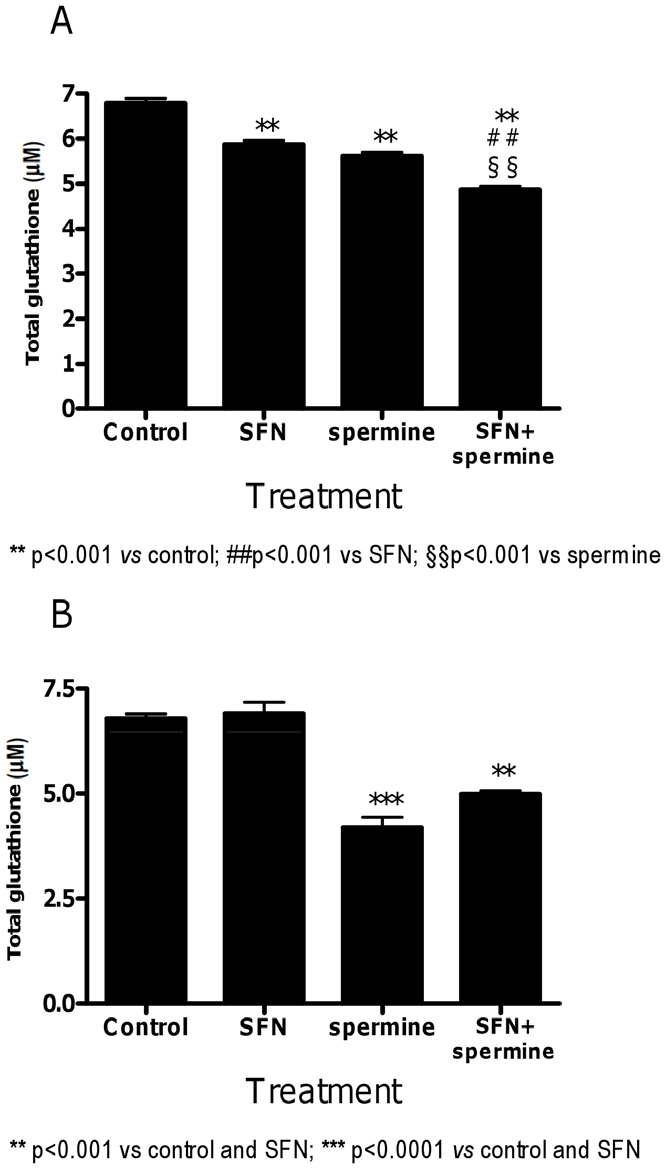
Effect of SFN and spermine on glutathione levels in Jurkat cells. Effect of SFN, spermine and SFN plus spermine on glutathione level determined by microplate fluorescence reader. Cells were treated for 3 h (A) or 24 h (B) with SFN (10 µM), spermine (0.5 mM) or SFN (10 µM)+spermine (0.5 mM). Results are means ± SEM of four independent experiments with triplicate dishes.

GSH depletion affects the antioxidant capacity of the coupled glutathione peroxidase–glutathione S-transferases system sensitizing target cells to oxidative stress. As shown in Figure 12A, short treatment with both SFN and spermine decreased cellular glutathione levels. The effect was significantly more marked in the cells treated with SFN plus spermine. However, the GSH-depletion induced by SFN was transient, as indicated by the levels of GSH similar to untreated cultures reported after 24 h of treatment (Figure 12B).

**Figure 13:**
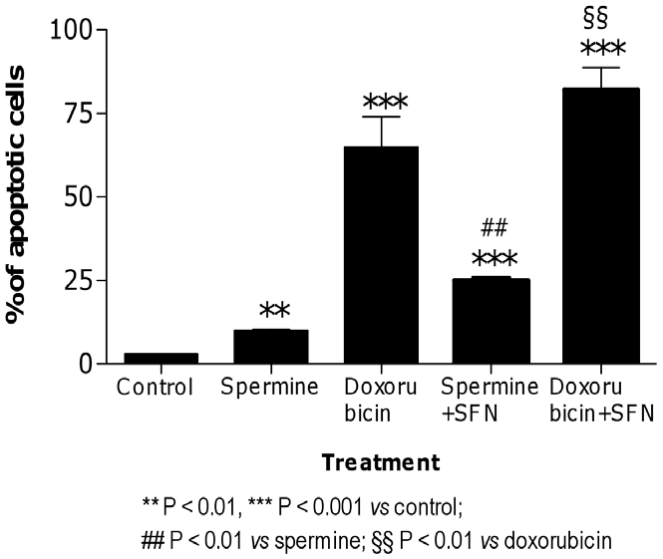
Induction of apoptosis by SFN plus spermine or doxorubicin in Jurkat cells. Fraction of apoptotic cells following pre- or co-treatment conducted with SFN (10 µM) and spermine (0.5 mM) or doxorubicin (0.01 mM), respectively. Results are means ± SEM of four independent experiments with triplicate dishes.

Finally, we analyzed the impact of RNA damage induction in the context of cell survival. In particular, the last part of our study was addressed to the study of the consequences and cellular handling of the RNA damage induced by doxorubicin and spermine alone and in combination with SFN. Flow cytometric analysis at 24 h post-spermine or -doxorubicin treatment showed that a significant proportion of cells underwent apoptosis, suggesting that this latter might be a relevant type of cell death in this exposure paradigm (Figure 13). To investigate whether SFN could increase the cytotoxicity of spermine and doxorubicin, cells were treated with a combination of spermine or doxorubicin plus SFN. The fraction of apoptotic cells induced after treatment with spermine or doxorubicin and pre- or co-treatment with SFN, respectively, was significantly greater than the fraction of apoptosis induced by exposure to spermine or doxorubicin alone (Figure 13).

## Discussion

In the present study, we clearly demonstrated that SFN did not alter RNA integrity in different cell systems. This is quite surprisingly because (1) our study also indicated that 3 h-treatment of cells with SFN was able to elicit intracellular ROS and different *in vitro* experiments reported that total RNA is susceptible to free radical attack owing to its single-stranded structure and the absence of protective histones [13], [14], [25]; (2) we previously demonstrated that SFN is able to induce DNA single strand breaks [9]. The reasons for these apparent conflicting results can be complex and, due to the still poor comprehension of the mechanisms leading to RNA damage, certainly requires *ad hoc* studies. However, Martinet et al. indicated that if the quantity of total RNA *per* cell is in the order of 10 pg, the intracellular free radical production needed to destroy the intracellular RNA content will be approximately 50 nM [14]. In our experiments, the concentrations of SFN used are lower than those used for the other compounds. They could be not enough to generate a level of ROS able to induce RNA damage in a cell system detectable by our capillary electrophoretic analysis. In addition, we reported that SFN-induced DNA damage depends entirely on intramitochondrial formation of ROS [9]: therefore, although it is a mere speculation deserving specific investigation, the site of ROS generation (i.e. mitochondrial *vs.* cytosolic or extracellular) might lie at the cross between the tendency of ROS to preferentially damage DNA, RNA or both. Such a hypothesis (i.e. that, unlike mitochondrially generated ROS, those formed extracellularly and within cytoplasm damage RNA) is indirectly strengthened by the finding that spermine and H_2_O_2_, which generate ROS at the extracellular and cytoplasmic level, do promote RNA damage (see below).

It is of worth to note that the treatment of RNA extracted from Jurkat cells with SFN 30 µM (a cell-free system) induced a significant RNA damage. These results cast light on the apparently unexpected inability of SFN to induce RNA damage in a cell system. Isothiocyanates compounds are normally unstable towards nucleophiles. Thus, it is conceivable that SFN interact with multiple biological nucleophiles such as cysteine in proteins and the tripeptide glutathione in a cell system [26], [27]. Modification of proteins is actually recognized as a key mechanism underlying the biological activity of isothiocyanates. *In vitro* experiments advocate the possibility of interaction between isothiocyanates and proteins having active thiol groups, such as thioredoxin and JNK phosphatase [28], [29], NOX2 [30], adenine nucleotide translocase [31], CYP isozyme [32], histone deacetylase [33], a transient receptor potential family of ion channels [34]. On the contrary, the only biological nucleophile available for SFN in our cell-free system is RNA. This means that, due to the high reactivity of SFN with cellular targets different from RNA, the amount of SFN able to react with RNA is low in a cell system and not enough to generate a level of RNA damage detectable by our capillary electrophoretic analysis.

The levels of ROS induced by SFN in Jurkat cells strongly decreased after 24 h of treatment with SFN. The dual effect of SFN on ROS levels can be associated with the dual effect of SFN on GSH levels. Over the last decade, a number of studies have shown that SFN is taken into cells almost entirely by conjugation with cellular GSH and is exported from the cell as a GSH conjugate [35], [36]. This can cause a decrease in cellular GSH, as we demonstrated in our cell system treated with SFN for 3 h. Later (24 h), SFN increases GSH levels by upregulating GSH synthesizing pathways mediated through γ-glutamylcysteine synthetase, the rate-limiting step in GSH synthesis. Thus, SFN may cause an immediate oxidizing effect, followed by an enhanced GSH synthesis and a return to a lower redox state [1].

Treatment of Jurkat cells with H_2_O_2_, SNAP, doxorubicin or spermine for 24 h induced RNA damage in all the cell lines tested and in normal human T lymphocytes. Under the selected exposure conditions, they did not alter cell viability by more than 60% compared to the concurrent control cultures. Since indirect mechanisms related to cytotoxicity may lead to enhanced RNA fragmentation, this finding implies that RNA damage induced by the above reported compounds is not the result of aspecific death-related events, but rather is likely to depend on their direct action on RNA.

In this context, it is important to note that changes of RNA size/level distribution observed in the RNA electropherograms do not necessarily indicate potential damages by a xenobiotic. Indeed – although the inclusion of RNase-free reagents and RNase inhibitors prevent RNA from damage by endogenous and exogenous RNAase – some processes such as RNA synthesis mechanisms and stabilizing/repairing mechanisms, may affect the RNA electropherograms. However, these are common problems with one of the most used genotoxicity assay, the Comet test. In the Comet assay, increased DNA migration is associated with increased levels of DNA damage such as strand breaks and alkali-labile sites. Furthermore, increased DNA migration may also derive from the presence of single-strand breaks associated with incomplete excision repair sites. On the other hand, a decrease in DNA migration can result from the ability of crosslinks to stabilize DNA molecule [37].

SFN did not affect the RNA damage induced by H_2_O_2_. This latter finding is not in keeping with our previous data, which reported that SFN was capable to afford protection against DNA damage induced by H_2_O_2_ [19]. These apparent contradictory results might largely depend on the different treatment conditions used in the two studies. Indeed a high H_2_O_2_ concentration (0.5 mM *vs.* 0.1 mM H_2_O_2_ in Ref. 19) was used throughout the present study. Such a higher dose of H_2_O_2_, selected to induce a significant RNA damage, is likely to yield a level of ROS that stoichiometrically overwhelms the scavenging potential of SFN. Also in support of our hypothesis is the inability of SFN at all concentrations studied (up to 100 µM) to scavenge H_2_O_2_, used at a very high concentration (7.5 mM), reported in a recent study [38].

Under our experimental conditions, the RNA damage induced by SNAP was not affected by SFN. SNAP exerts its toxic activity through the production of reactive nitrogen species [39]. An aspect worth considering is that SNAP could generate radicals that are not SFN-sensitive. Other experiments are necessary for supporting this hypothesis.

Pre-treatment and co-treatment with SFN markedly enhanced the RNA-damaging activities of doxorubicin, one of the most effective anticancer drugs ever developed. Doxorubicin undergoes bioreductive activation by redox-cycling reactions. One-electron addition to the quinone moiety of doxorubicin is associated with the formation of a semiquinone. The latter quickly regenerates its parent quinone by reducing ground state oxygen to ROS, such as superoxide anion and its dismutation product, hydrogen peroxide. Toxicity of doxorubicin rests with the DNA intercalation of the semiquinone radical. The superoxide generated in this redox-cycling process induces additional, qualitatively different, DNA lesions [40].

The biological role of thioredoxin reductase is to transfer reducing equivalents from NADPH to various oxidized substrates. Thioredoxin reductase, together with its substrate thioredoxin, forms a redox system, which plays multiple roles and acts on different substrates such as lipoic acid, lipid hydroperoxides, vitamin K3 and ascorbyl free radicals [41]. The mammalian thioredoxin system protects cells and tissues against oxidative stress. Thioredoxin is a scavenger of hydroxyl radical and a quencher of singlet oxygen. However, it does not scavenge superoxide anion [42]. Previous studies have shown that doxorubicin is a substrate for mammalian thioredoxin reductase. In particular, Ravi and Das demonstrated that the *E. coli* thioredoxin system enhanced the redox-cycling of anthracyclines and increased the generation of superoxide anion [43].

In keeping with a previous study [44], we demonstrated that SFN induced the thioredoxin reductase activity. The increase in thioredoxin reductase activity by SFN was observed at 24 h and at a time-point before the 24 h, i.e. 3 h. This suggests that the increase in thioredoxin reductase activity by SFN could be the key mechanism involved in the enhancement of doxorubicin-induced RNA damage by SFN in both treatment protocols. Depletion of intracellular GSH caused by SFN, to form the SFN-SG conjugate, is a fundamental step in the modulation of thioredoxin reductase expression [43]. When cells were treated with SFN for 3 h, we observed an initial decrease in cellular GSH levels. A recovery of intracellular GSH levels was reported after a longer treatment (24 h). Interestingly, the thioredoxin reductase activity was still elevated at 24 h of treatment with SFN. Hence, possibly what SFN may be promoting is increasing the cellular damage of doxorubicin by modulating its bioreductive activation. A depletion of GSH does not appear to be required for the thioredoxin reductase activation.

The polyamine spermine has an important role in the viability and propagation of most cells. However, many studies reported that spermine is also the source of cytotoxic metabolites Amine oxidase in fetal calf serum catalyses the oxidative deamination of spermine and produces aminodialdehyde, H_2_O_2_, and ammonia. Aminodialdehyde produced during the oxidation of spermine subsequently undergoes spontaneous β-elimination to form acrolein [44]. Notably, the degree of cytotoxicity of spermine is nearly parallel with the amount of acrolein produced and spermine toxicity is prevented by aldehyde dehydrogenase, which eliminates these reactive species and prevents the formation of acrolein [45].

In our experimental settings, spermine induced a significant RNA damage, which was clearly potentiated by pre-treatment with SFN, while it remained unchanged in the co-treatment protocol.

Interestingly, the RNA-damaging effects observed for spermine might directly depend on the formation of ROS promoted by spermine metabolism. Therefore, we performed a set of experiments aimed at determining whether and how much spermine gives rise to ROS under the treatment conditions adopted for evaluating RNA damage. Here the formation of ROS was directly monitored in experiments involving the sensitive probe DCFH, which – upon oxidation – is converted to its fluorescent derivative, DCF. Surprisingly, we did not observe an increase in the levels of ROS after 24 h-treatment with spermine or SFN plus spermine. However, when we analyzed the levels of ROS after 3 h of treatment, we recorded a significant increase in the ROS levels both in cells treated with spermine and in cells treated with the association. The highest levels of ROS observed after treatment with spermine easily explain their ability to attack RNA and initiate RNA damage. Despite the lack of a ROS increase after 24 h of treatment with spermine, we recorded RNA damage after 24 h of treatment with spermine. In this context, it is important to note that Sharmin et al. [45] demonstrated that the stability of acrolein in the presence of fetal calf serum is strongly decreased with time. The remaining acrolein produced from spermine decreased from about 60% to about 10% in 90 min. This observation can easily explain the low levels of ROS we observed after 24 h treatment with spermine. The persistence of RNA damage can be due to the fact that, although cells may have multiple mechanisms of dealing with RNA damage [46]–[48], glycosylases - able to remove oxidatively damaged bases - have not yet been identified in RNA. Along this line, a previous attempt to induce chain breaks at RNA through the use of 7,8-dihydro-8-oxo-2′-deoxyguanosine specific glycosylase failed [49]. Our results strongly suggested that spermine induced RNA damage through the production of ROS in the first hours of treatment and that RNA damage persisted due to the lack of specific repair systems. This hypothesis was confirmed by an additional analysis of RNA integrity after 6 h of cell treatment with spermine, where a decrease in the RIN value was detected compared to the concurrent control cultures (data not shown).

As regards the effect of SFN on the RNA damage induced by spermine, pre-treatment with SFN markedly enhanced the RNA-damaging potential of spermine. In an attempt to delineate the mechanism involved in this effect, we decided to investigate the levels of ROS induced by SFN plus spermine at 3 h of treatment. We recorded a statistically significant increase in the ROS level after the combined treatment (pre-treatment with SFN for 3 h, followed by treatment with spermine for 3 h). The increase in ROS production caused by SFN plus spermine was additive. This is not unexpected because other papers have shown that SFN causes a transient increase in ROS production [50]. SFN is also able to inhibit and reduce the expression of aldehyde dehydrogenase [51], [52], an enzyme known to prevent and/or eliminate the aldehydes produced during the oxidation of spermine [45]. However, a 3 h-treatment with SFN is too short to lead to enzyme induction/downregulation and it is unlikely that enzyme downregulation is involved in the enhancement of RNA-damaging potential of spermine by SFN.

As to the co-treatment conditions, SFN did not affect the RNA-damaging activity of spermine. Recent studies reported that spermine [53] and SFN [36] exposure reduced intracellular GSH levels. Data presented here lend further support to this latter notion and extend its toxicological meaning. Our results indeed demonstrated that spermine and SFN reduced intracellular GSH levels and that the decrease in the GSH levels was more pronounced in the cultures co-treated with SFN plus spermine. As reported above, GSH represents the major driving force for SFN accumulation by undergoing conjugation with the entering SFN [35]. Moreover, Zhang demonstrated that altering cellular GSH levels results in proportional changes in cellular SFN uptake and accumulation [36]. Along this line, the marked reduction of GSH evidenced in the cells co-treated with spermine plus SFN can hamper the uptake of SFN by GSH thereby strongly reducing or almost suppressing the intracellular levels of SFN. In this condition, SFN could not affect spermine toxicity.

Notably, the effects of SFN on the RNA damaging activity of doxorubicin, spermine, SNAP and H_2_O_2_ lacked cell-type specificity, since it could be observed in HL-60, KU812F and normal T cells.

As to the cytotoxic relevance of the events described in this study, we found that the treatment of cultures with spermine or doxorubicin, both alone and more strongly in combination with SFN, induced apoptosis. However, it is hard to distinguish between the relative importances of RNA damage as compared to other possible toxic effects promoted by the above reported chemicals. A lot of studies reported proapoptotic activity of SFN in many experimental models [2], [4], [9], [54]. In our study, SFN did not induce RNA damage. Thus it is conceivable that the presence of RNA damage might simply concur to the overall toxic response induced by a chemical agent in targeted cells. Various cell signaling network models indicate that partial inhibition of a number of targets is more effective than the complete inhibition of a single target in many areas of medicine [55]. In this light, our data also pave the way to an appraisal of the contribution of spermine- or doxorubicin-induced RNA damage to the net cytotoxic response of intoxicated cells.

In conclusion, we demonstrated that RNA did not represent a target for the toxic action of SFN. Furthermore, SFN was unable to protect cells from RNA insult induced by different toxic agents.

The induction of RNA damage still represents a maturing approach and its true potential remains to be defined and studied. As an example, an open question emerged from the present study which would deserve specific studies deals with the problem of RNA differential susceptibility to ROS arising from selected subcellular compartments. However, it is worth noting that RNA is being investigated as a potential target for new anticancer agents: the induction of damage to RNA is clearly an interesting and potentially useful therapeutic approach, as demonstrated by successful pre-clinical and clinical trials [56]–[59]. In this context, the induction of RNA damage may represent an additional target of doxorubicin. The ability of SFN, at nutritionally attainable concentrations, to enhance RNA damage and cytotoxicity deserves consideration as an additional mechanism potentially responsible for the potentiating effects of SFN associated with conventional anticancer drugs, such as doxorubicin. Notably, polyamine analogues have demonstrated interesting pre-clinical results in different model systems of cancer, but their clinical utility has been limited by apparent toxicity [60]. The use of polyamines in association with SFN, along with reducing the dosage of polyamines, may enhance its anticancer efficacy.
