# Correction: Sulforaphane Potentiates RNA Damage Induced by Different Xenobiotics

**DOI:** 10.1371/annotation/d198fded-586e-4f8a-9f4a-598eb0c81547

**Published:** 2012-05-15

**Authors:** Carmela Fimognari, Monia Lenzi, Piero Sestili, Eleonora Turrini, Lorenzo Ferruzzi, Patrizia Hrelia, Giorgio Cantelli-Forti

There was an error in the published funding statement. The correct funding is: This work was supported by Ministero dell'Università e della Ricerca (http://sitouniversitario.cineca.it; grant number: 200974K3JC). The funder had no role in study design, data collection and analysis, decision to publish, or preparation of the manuscript.

